# [^125^I]INFT: Synthesis and Evaluation of a New Imaging Agent for Tau Protein in Post-Mortem Human Alzheimer’s Disease Brain

**DOI:** 10.3390/molecules28155769

**Published:** 2023-07-31

**Authors:** Roz R. Limpengco, Christopher Liang, Yasmin K. Sandhu, Jogeshwar Mukherjee

**Affiliations:** Preclinical Imaging, Department of Radiological Sciences, University of California-Irvine, Irvine, CA 92697, USA; rlimpeng@uci.edu (R.R.L.);

**Keywords:** [^125^I]INFT, [^125^I]IPPI, post-mortem human Tau, Alzheimer’s disease, neurofibrillary tangles, autoradiography

## Abstract

Aggregation of Tau protein into paired helical filaments causing neurofibrillary tangles (NFT) is a neuropathological feature in Alzheimer’s disease (AD). This study aimed to develop and evaluate the effectiveness of a novel radioiodinated tracer, 4-[^125^I]iodo-3-(1H-pyrrolo[2,3-c]pyridine-1-yl)pyridine ([^125^I]INFT), for binding to Tau protein in postmortem human AD brain. Radiosynthesis of [^125^I]INFT was carried out using electrophilic destannylation by iodine-125 and purified chromatographically. Computational modeling of INFT binding on Tau fibril was compared with IPPI. In vitro, autoradiography studies were conducted with [^125^I]INFT for Tau in AD and cognitively normal (CN) brains. [^125^I]INFT was produced in >95% purity. Molecular modeling of INFT revealed comparable binding energies to IPPI at site-1 of the Tau fibril with an affinity of IC_50_ = 7.3 × 10^−8^ M. Binding of [^125^I]INFT correlated with the presence of Tau in the AD brain, confirmed by anti-Tau immunohistochemistry. The ratio of average grey matter (GM) [^125^I]INFT in AD versus CN was found to be 5.9, and AD GM/white matter (WM) = 2.5. Specifically bound [^125^I]INFT to Tau in AD brains was displaced by IPPI (>90%). Monoamine oxidase inhibitor deprenyl had no effect and clorgyline had little effect on [^125^I]INFT binding. [^125^I]INFT is a less lipophilic imaging agent for Tau in AD.

## 1. Introduction

**Figure 1:**
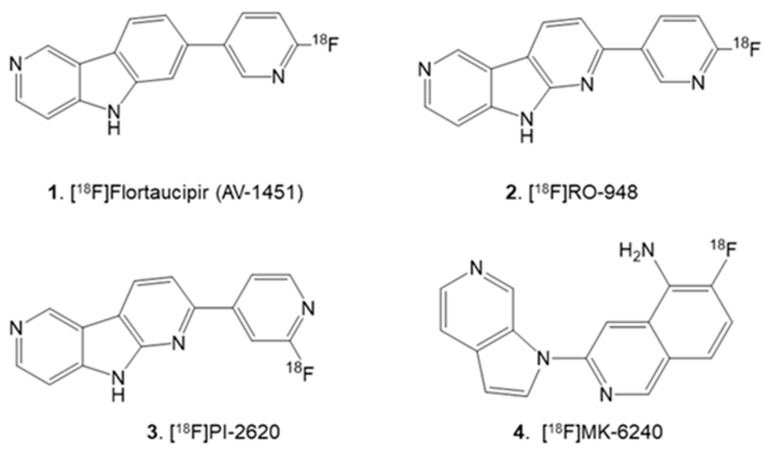
PET imaging agents for Tau in human use: Pyrrole Derivatives: **1**. [^18^F]Flortaucipir or [^18^F]AV-1451; **2**. [^18^F]RO-948; **3**. [^18^F]PI-2620; **4**. [^18^F]MK-6240 (Azindole Derivative).

**Figure 2:**
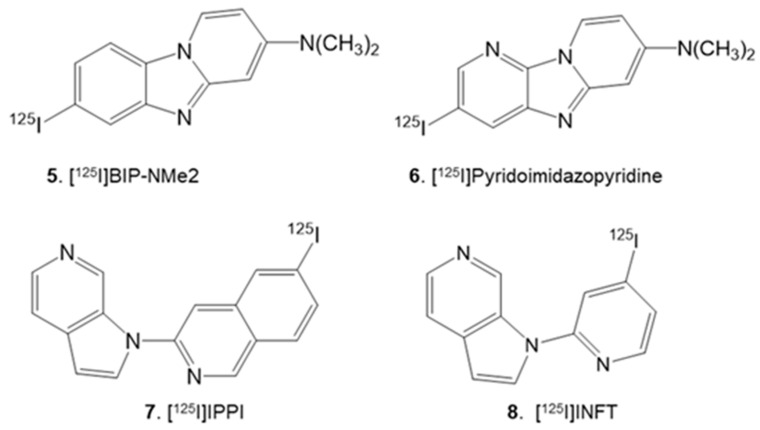
Radioiodinated Tau binding radioligands under development: Imidazole derivatives: **5**. [^125^I]BIP-NMe_2_; **6**. [^125^I]Pyridoimidazopyridine; Azindole derivatives: **7**. [^125^I]IPPI and [^124^I]IPPI; **8**. [^125^I]INFT (reported here).

**Figure 3:**
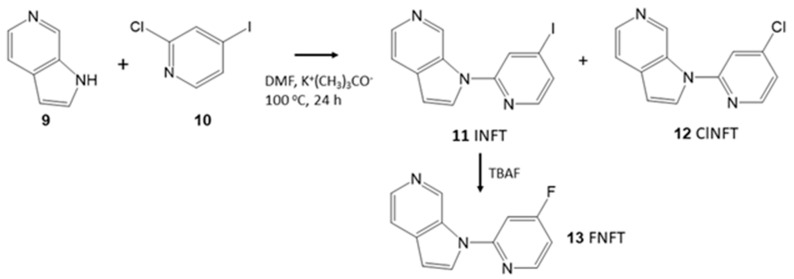
Synthesis of INFT and Analogs: Azaindole **9** reacted with 2-chloro-4-iodopyridine **10** in dimethylformamide (DMF) and potassium *tert*-butoxide (K^+^(CH_3_)_3_CO^−^. Reaction products included INFT **11** and ClNFT **12**. INFT was reacted with tetrabutylammonium fluoride (TBAF) in tetrahydrofuran to provide FNFT **13**.

**Figure 4:**
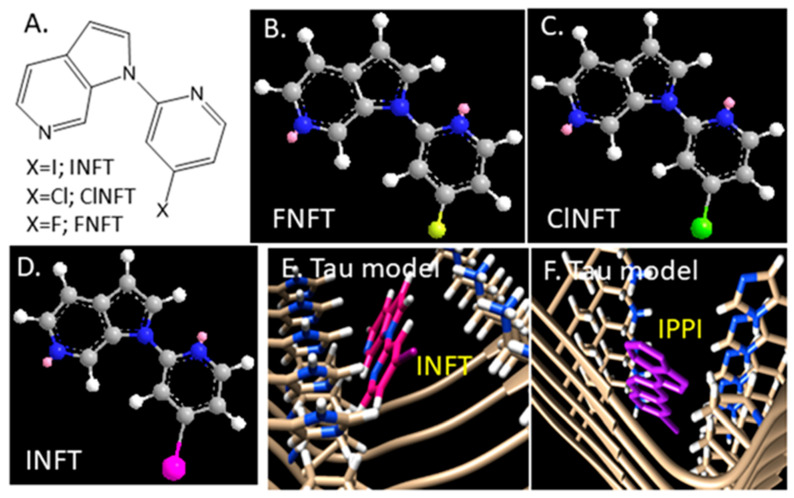
INFT in Tau Molecular model: (**A**) Chemical structures of azindole derivatives (INFT, ClNFT and FNFT). (**B**–**D**) Energy minimized structures of FNFT.mol (**B**) ClNFT.mol (**C**) and INFT.mol. (**D**) for use in Auto-docking studies. (**E**) Chimera AD Tau model showing binding of INFT (in red) at Tau binding Site 1. (**F**) Chimera AD Tau model showing binding of IPPI (in purple) at Tau binding Site 1 (Atom colors Blue: Nitrogen; Yellow: fluorine; Green: Chlorine; Pink: Iodine).

The aggregation of Tau protein into paired helical filaments causing neurofibrillary tangles (NFT) is a neuropathological feature in Alzheimer’s disease (AD) [1]. Efforts have been underway on developing and using Tau PET imaging agents since they can play an essential role in clinical studies to evaluate disease progression [2,3]. Pyrrole derivatives such as [^18^F]T807 (Figure 1, **1**) [4] [^18^F]RO6958948 (Figure 1, **2**) [5] and [^18^F]PI-2640 (Figure 1, **3**) [6] are being used for human Tau PET imaging in AD. Although off-target MAO-B binding has been raised as a concern for the pyrrole derivatives, in vivo, PET data are assumed to be free of this off-target binding [7]. The next generation of Tau PET radiotracer, [^18^F]MK-6240 (Figure 1, **4**) [8,9], based on azaindole structure without the MAO-B off-target binding concerns, is now being used in PET studies [10]. Recent PET studies show a significant amount of off-target binding of [^18^F]MK-6240 in the meninges of AD subjects which may confound the measurement of cortical Tau [11].

**Figure 5:**
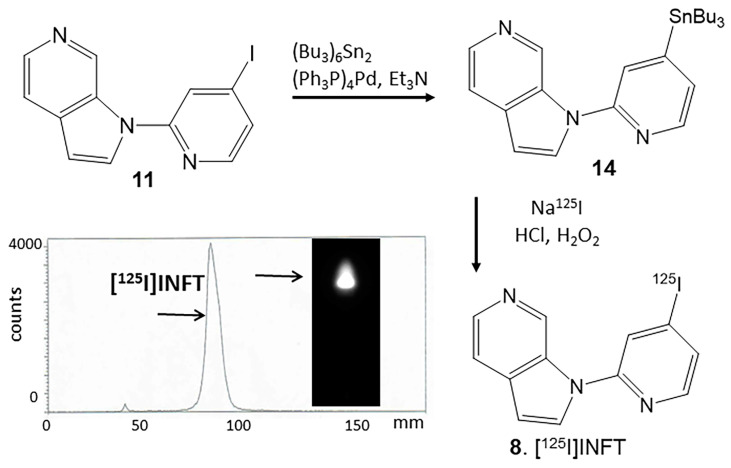
Radiosynthesis of [^125^I]INFT: INFT, **11** was refluxed with bis(tributyltin) in the presence of tetrakis(triphenylphosphine)palladium(0) for 24 h to provide tributyltin precursor, **14**. Tin precursor **14** was reacted with sodium [^125^I]iodide under oxidative conditions using hydrogen peroxide to provide [^125^I]INFT, **8**. The radioactive thin layer chromatogram of [^125^I]INFT shows a predominant peak with purity of >95%.

**Figure 6:**
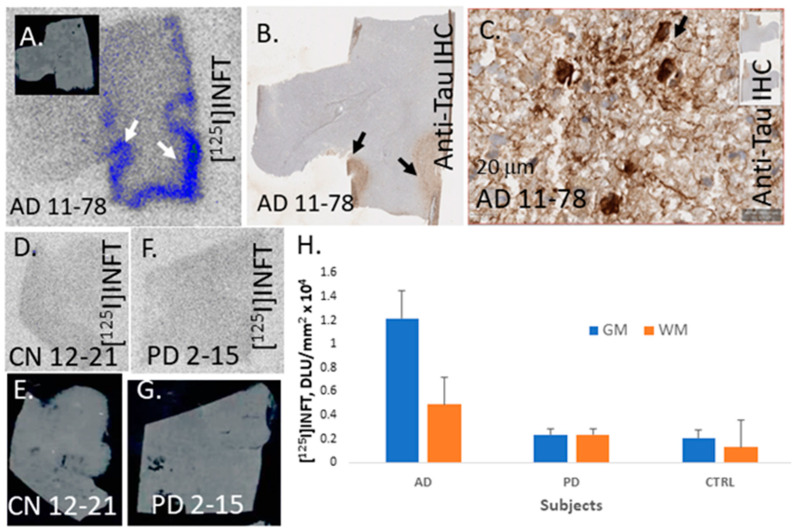
Postmortem human brain autoradiography [^125^I]INFT: (**A**) Human postmortem AD brain anterior cingulate (10 μm thick sections) showing grey matter (GM) and white matter (WM). Binding of [^125^I]INFT to Tau (indicated by arrows) in AD 11–78. (**B**) Anti-Tau staining of AD 11–78 showing the presence of NFT in GM regions. (**C**) Magnification of GM region shows NFT (arrow). (**D**,**E**) Cognitively normal subject (CN) (10 μm thick sections) with no [^125^I]INFT binding in GM. (**F**,**G**) Parkinson’s disease (PD) brain (10 μm thick sections) with no [^125^I]INFT binding in GM. (**H**) Plot of [^125^I]INFT binding to GM and WM in AD, PD and CN (Ctrl) subjects.

Progress in the development of radioiodinated imaging agents for Tau has been relatively slow. Recently, a series of radioiodinated imidazo derivatives (e.g., Figure 2, **5**, **6**) have been developed, and in vitro, studies in postmortem AD brains have been reported [12,13]. The binding of these agents to Tau in the AD brain was prominent, and in vivo brain uptake in normal mice appeared suitable. Off-target binding to MAO-B of these radioiodinated derivatives has not been reported. We have previously reported azaindole derivatives [^125^I]IPPI [14] and [^124^I]IPPI [15] as radioiodinated analogs of [^18^F]MK-6240 for Tau imaging. Selective binding of [^124/125^I]IPPI to Tau was observed in the anterior cingulate of postmortem human AD brains. The binding of [^124/125^I]IPPI was quantitatively correlated with the Tau load measured by anti-Tau immunohistochemistry of the same subjects [15]. Off-target binding of [^124/125^I]IPPI to MAO-A or MAO-B would not be a concern because of a lack of effect of clorgyline (MAO-A) or deprenyl (MAO-B) on [^125^I]IPPI binding in the AD brains [14].

Our previous work with [^124/125^I]IPPI confirmed the delineation of Tau in the human AD postmortem brain, and they appear to be sensitive to different levels of Tau. They may suggest their ability to differentiate the stage of the disease [14,15]. For in vivo preclinical evaluation, transgenic AD mice models play an essential role in evaluating proteinopathy imaging agents. Although differences may occur between the protein aggregates found in transgenic AD mice and AD humans, when found to be similar, they can play a significant role in imaging agent development and potential therapeutics evaluation. We have recently reported the successful evaluation of [^124^I]IBETA binding to Aβ plaques in 5xFAD transgenic AD mice, both in vitro and by in vivo PET/CT [16]. This is a good example of using transgenic AD mice models for imaging agent development headed for translational studies to humans. Similar efforts for evaluating Tau imaging agents in AD transgenic mice have been pursued [17]. Although transgenic mice expressing Tau are available, [18] the ability to use them for imaging agent evaluation of Tau agents currently in human use continues to evolve.

For optimal in vivo imaging, a lipophilicity log P of approximately 2 is preferred. It enables optimal brain uptake across the blood-brain barrier and minimizes nonspecific binding by relatively faster clearance from non-target brain regions. These attributes can provide higher target-to-nontarget ratios, thus improving the properties of the imaging agent. The reported Log D of [^18^F]MK-6240, the clinically used PET imaging agent for Tau, is 3.32 (Table 1) [8,10]. This is higher than the optimal lipophilicity for in vivo imaging agents. The calculated lipophilicity of [^125^I]IPPI is significantly higher (log P = 4.34), compared to [^18^F]MK-6240 and may lead to high levels of nonspecific binding in vivo.

To reduce lipophilicity, we examined the replacement of the iodoisoquinoline ring in [^125^I]IPPI **7** with a smaller iodopyridine ring. It should be noted that [^18^F]MK-6240 and [^125^I]IPPI are second-generation Tau imaging agents developed to avoid off-target binding to MAO-B. Our previous findings have shown that [^125^I]IPPI binding to human AD brain slices is unaffected by MAO-A and MAO-B inhibitors [14]. To what extent the isoquinoline ring in [^18^F]MK-6240 and [^125^I]IPPI helps avoid MAO binding remains to be seen. Changing the iodoisoquinoline ring to the iodopyridine ring would remove the benzene ring and thus reduce the log P significantly. However, the effect of this change on both the binding affinity to Tau and the lack of binding to MAO-A and MAO-B remains to be determined.

Thus, we prepared 4-iodo-2-(1H-pyrrolo[2,3-c]pyridine-1-yl)pyridine ([^125^I]INFT; Figure 2, **8**), which has a calculated log P of 2.96. This reduced lipophilicity of [^125^I]INFT compared to [^125^I]IPPI will be useful for in vivo studies in reducing the nonspecific binding, provided it maintains a high affinity for Tau and lacks binding to MAO-A and MAO-B. Here we report: (1) Synthesis of INFT **11** and the chlorinated (ClNFT **12**) and fluorinated (FNFT **13**) analogs (Figure 3); (2) Molecular docking studies to human Tau; (3) Radiosynthesis of [^125^I]INFT; (4) Measurement of binding affinity of the analogs for Tau using postmortem AD brain; (5) Autoradiographic studies in postmortem AD, Parkinson’s disease (PD) and cognitively normal (CN) brain sections, and (6) Drug effects (MAO-A and MAO-B) on the binding of [^125^I]INFT in postmortem AD brains.

## 2. Results

Synthesis of INFT **11** was carried out in a single step by reacting azaindole **9** with 2-chloro-4-iodopyidine **10** (Figure 3). The nucleophilic displacement reaction resulted in displacing either the iodine or chlorine, thus providing INFT **11** as well as ClNFT **12** in the product mixture. Additionally, some product as a result of azaindole displacing both iodine- and chlorine- in the same molecule was also observed (structure not shown). In modest yields, INFT and ClNFT were isolated as pure products by preparative chromatography. For preparing the fluorinated derivative, FNFT **13**, nucleophilic displacement of iodine in INFT was carried out using tetrabutylammonium fluoride (Figure 3).

We used our previously reported procedures of Chimera-AutoDock to assess the binding of INFT, ClNFT and FNFT (Figure 4A) to the cryo-EM three-dimension structure of Tau fibril [14,15]. Energy-minimized molecular models of INFT, ClNFT and FNFT were made using Chem Draw 3D (Figure 4B–D). In our previous findings with IPPI binding to the Tau fibril in AD, four binding sites were identified [14]. Comparing the binding energy values (Kcal/mol) at the four sites for IPPI, the lowest energies for INFT were found for Site 1. Docking studies with INFT revealed preferential binding at Site 1 (Figure 4E) and was similar to the binding of IPPI to Site 1 shown in Figure 4F. The binding energies of INFT to Sites 2–4 were weaker when compared with Site 1 and weaker than the binding energies of IPPI for these sites. The lack of the second phenyl ring in INFT (compared to isoquinoline in IPPI) potentially reduces the hydrophobic interactions, thus weakening the binding energies. Compared to INFT, binding energies of ClNFT and FNFT were weaker at Site 1. The larger iodine atom compared to the smaller chlorine and fluorine (Figure 4B–D) significantly reduces hydrophobicity and weakens the binding. Interestingly, the isoquinoline analog of FNFT, with the additional aromatic ring, has a significant affinity for Tau [9,14].

Sodium iodide [^125^I]NaI (ARC Inc., St. Louis, MO, USA) was used to prepare the electrophilic substitution of the tributyltin derivative using our previously reported radioiodination methods. [16,19]. The same reaction was used to synthesize [^125^I]INFT from the tributyltin derivative **14** (0.1 mg in 0.1 mL ethanol) and 3.4 MBq [^125^I]NaI. The reaction was allowed to proceed at room temperature for 60 min before it was terminated by adding sodium bisulfite.

The purification and isolation of [^125^I]INFT were conducted on preparative TLC. Two rounds of extraction were performed using dichloromethane. The extract was then dried using anhydrous MgSO_4_. Radio TLC confirmed a radiochemical purity of >95% [^125^I]INFT (Figure 5). Using the molar activity of no-carrier added [^125^I]sodium iodide, the molar activity of [^125^I]INFT was estimated to be approximately 90 TBq/mmole under the no-carrier added conditions. No other major radiolabeled organic side products were observed successfully substituted with tributyltin substituent in 25% yield, sufficient for use in radiolabeling procedures.

Lipophilicity of radiolabeled [^125^I]INFT and [^125^I]IPPI were measured by partitioning between 1-octanol and PBS buffer. Log D of [^125^I]INFT was measured to be 2.71, comparable to the calculated value of 2.96 (Table 1). As expected for [^125^I]IPPI, the measured value was 4.10, comparable to the calculated value of 4.34. The reported log D of the clinically used human PET radiotracer, [^18^F]MK-6240 is 3.32 [8]. Thus, [^125^I]INFT is less lipophilic is less lipophilic compared to [^18^F]MK-6240.

In vitro binding affinity of the unlabeled compounds was evaluated in AD brain slices labeled with [^125^I]INFT. The anterior cingulate of the subjects were first evaluated for the presence of Tau using [^125^I]IPPI, and as expected, all AD subjects showed the presence of Tau, as reported previously [14]. Assay conditions using [^125^I]INFT were similar to our reported procedures using [^125^I]IPPI. Different compounds (Table 1) at concentrations 10^−9^ M to 10^−5^ M were used for the competition assay with [^125^I]INFT. Incubation of the brain slices with the respective compounds and [^125^I]INFT were done in 10% alcohol in PBS buffer using the reported procedures.[14] Binding of [^125^I]INFT to Tau in brain slices was quantified in Digital Light Units (DLU)/mm^2^ using the Optiquant image analysis program. Data were analyzed using the following procedure: (1) the non-specific binding of [^125^I]INFT was subtracted for all samples; (2) the specific binding was normalized to 100% (no competitive ligand) and (3) the binding isotherms were fit to the Hill equation (KELL BioSoft software (v 6), Cambridge, UK) to provide inhibitor concentration (IC_50_) which is the inflection point of the isotherm where 50% of the [^125^I]INFT binding to Tau. Table 1 shows the IC_50_ values of the four compounds tested and the reported [^18^F]MK-6240 [8]. The affinity of INFT and IPPI was similar, while ClNFT and FNFT were weaker. It should be noted that the affinity of both INFT and IPPI measured by competition with [^125^I]INFT most probably reflects binding to Site 1 (Figure 4E,F) based on the Tau model study. Unlike INFT, IPPI also has a significant binding ability to additional Tau Sites (Sites 2,3,4; [14]), which may account for the difference in affinity when a [^3^H]isopquinoline derivative was used for measuring the binding affinity of IPPI [9].

Binding of [^125^I]INFT was evaluated in six AD subjects, 6 cognitively normal (CN) and 6 PD subjects. Figure 6A shows the binding of [^125^I]INFT to the GM regions of one of the AD subjects (AD 11–78). Low levels of nonspecific binding were observed in the WM regions. The adjacent brain section of AD 11–78 was Immunostained for total Tau, shown in Figure 6B. Areas of Tau IHC in Figure 6B corresponded to [^125^I]INFT binding in Figure 6A. A closer view of IHC in AD 11–78 in Figure 6C confirmed the presence of NFT. Brain slices from CN subjects (CN 12–21) are shown in Figure 6D,E with non-selective nonspecific binding of [^125^I]INFT in the GM and WM. Similarly, brain slices from PD subject (PD 2–15) in Figure 6F,G did not show any selective GM binding of [^125^I]INFT. The absence of Tau was confirmed with anti-Tau IHC in the CN and PD subjects. The average binding of [^125^I]INFT in the three groups of subjects is shown in the plot in Figure 6H. Both CN and PD subjects exhibited lower GM binding of [^125^I]INFT than that in AD, which was consistent with the absence of Tau. Binding of [^125^I]INFT in AD WM was also higher than in CN and PD subjects. The average GM/WM [^125^I]INFT ratio in AD was 2.48, AD GM/CN GM was 5.9 and AD GM/PD GM = 5.14. The same AD subjects have been previously shown to exhibit significant amounts of [^18F^]Flotaza binding to Aβ plaques [15,20].

**Figure 7:**
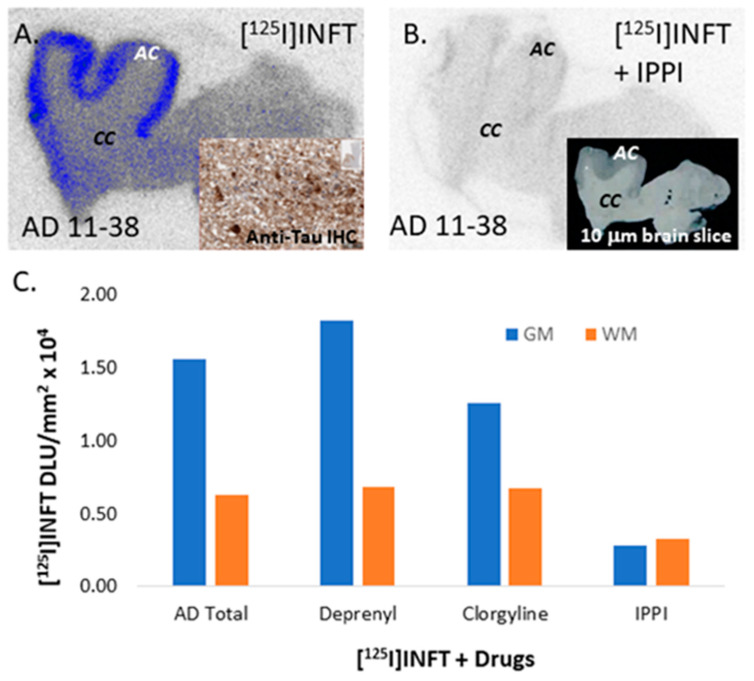
Competition of [^125^I]INFT with Drugs: (**A**) Postmortem human brain 10 μm thick sections (AD subject 11–38) showing [^125^I]INFT binding to grey matter, AC with low nonspecific binding in white matter, CC. Inset shows immunostained 11–38 with anti-Tau Dako A0024 for total Tau. (**B**) IPPI 10 μM displaced [^125^I]INFT binding from the GM (inset shows a scan of the brain slice). (**C**) Plot comparing GM and WM in AD subjects with different drugs (Deprenyl for MAO-B, Clorgyline for MAO-A and IPPI for Tau).

In the absence of other drugs, significant [^125^I]INFT binding was observed in the AD GM comprising of AC, while the WM consisting of CC has little binding (AD 11–38; Figure 7A). The ratio of GM (AC) to WM (CC) was >5 for AD 11–38 (Figure 7A), while the average GM/WM = 2.48. The binding of [^125^I]INFT correlated very well with immunohistochemical findings of total Tau, as seen in Figure 7A inset, thus confirming the binding of [^125^I]INFT to regions with Tau in the brain slice. Upon the addition of unlabeled IPPI (10 μM), most of the binding of [^125^I]INFT to AC was abolished (Figure 7B), as expected. The ratio, GM/WM ≤ 1, suggests that [^125^I]INFT binding was at the same Tau binding site as IPPI. Since off-target binding of some of the reported Tau imaging agents to monoamine oxidases have been reported [14], we performed competition experiments of [^125^I]INFT with (*R*)-deprenyl for potential binding to MAO-B and clorgyline for potential binding to MAO-A [21]. There was no decrease in the binding of [^125^I]INFT in the presence of 10 μM (*R*)-deprenyl (GM/WM = 2.68; Figure 7C), suggesting a lack of MAO-B binding by [^125^I]INFT. This is similar to our previous observations on the lack of any effect of (*R*)-deprenyl on the binding of [^125^I]IPPI to Tau [14]. In the presence of 10 μM clorgyline, the [^125^I]INFT ratio was reduced, GM/WM = 1.88 (Figure 7C), suggesting approximately 24% of [^125^I]INFT was displaced. Thus, at a high concentration of clorgyline, a fraction of [^125^I]INFT binding is affected.

## 3. Discussion

Radiolabeled [^125^I]INFT is a new iodinated imaging agent for Tau. The binding of [^125^I]INFT to Tau in AD brains was confirmed by correlation with anti-Tau immunostained sections and drug challenges, including IPPI. Because of the truncated aromatic ring structure of INFT, it is significantly less lipophilic compared to our previously reported [^125^I]IPPI. Due to its lower lipophilicity, it suggests that for in vivo imaging studies, nonspecific binding of [^125^I]INFT may be lower than [^125^I]IPPI. Thus, iodine-124 analog, [^124^I]INFT may have potential value for in vivo applications for PET studies in AD mice models [16,22]. Both [^125^I]INFT and [^125^I]IPPI provide reasonable GM/WM ratios in AD postmortem brains, although ratios with [^125^I]IPPI are higher [14,15]. The availability of radioiodinated Tau imaging agents will enable in vitro evaluation of drugs [23,24].

Based on the structural features of the limited series of compounds, it may be surmised that the iodopyridine ring in INFT and the iodoisoquinoline ring of IPPI bind in a hydrophobic pocket. The larger iodine atom may be preferred because of the size of this pocket and enhanced hydrophobic interactions. Replacing the iodine with fluorine marginally lowered the affinity in the case of INFT, whereas replacing the iodine with fluorine in the case of IPPI had little effect. [14] Tau molecular models suggest that INFT may be more selective in binding to one particular site (Site 1, Figure 4), whereas IPPI was shown to have reasonable binding energies at four different Tau sites, similar to MK-6240 [14]. Thus, there may be subtle differences between [^125^I]INFT binding to Tau versus that of [^125^I]IPPI binding to Tau. Further studies are underway to evaluate [^125^I]INFT binding in other Taupathies, such as Picks disease and corticobasal degeneration (CBD).

Radiolabeled fluorine-18 analog of FNFT may be prepared for evaluation using our previously reported methods of [^18^F]-nucleophilic substitution on pyridine rings [25]. However, although FNFT has the lowest lipophicity in the series (clogP 2.05) and is similar to some of the optimal fluorinated imaging agents developed [26], it may not be a suitable in vivo Tau PET imaging agent due to its lower affinity for Tau. Other potential fluorinated analogs of INFT would be to replace the iodine with a trifluoromethyl group or a fluoroalkyl group. Fluorine-18 trifluoromethyl derivatives have been previously developed as PET imaging agents [27]. Fluorine-18 labeled fluoropropyl substitutions in pyridine derivatives have been used as successful PET radiotracers [28].

## 4. Materials and Methods

### 4.1. General Methods

General methods were similar to those described previously [14,16,19]. Iodine-125 sodium iodide was purchased from American Radiolabeled Chemicals, Inc., St. Louis, MO, USA (iodine-125 sodium iodide, carrier-free (specific activity = 643 MBq/μg) in 0.01 N NaOH). Iodine-125 radioactivity was counted in a Capintec CRC-15R dose calibrator, while low-level counting was carried out in a Capintec Caprac-R well-counter. RadioTLC was scanned on an AR-2000 imaging scanner (Eckart & Ziegler, Berlin, Germany). Electrospray mass spectra were obtained from a Model 7250 mass spectrometer (Micromass LCT). Proton NMR spectra were recorded on a Bruker OM EGA 500-MHz spectrometer.

### 4.2. Synthesis

4-Iodo-2-(1H-pyrrolo[2,3-c]pyridine-1-yl)pyridine (INFT), **11**: 6-Azaindole, Figure 3, **9** (102 mg, 0.86 mmol) was treated with sodium tert-butoxide (99 mg, 1.03 mmol) in dimethylformamide (DMF, 1 mL) for 15 min at 100 °C. Subsequently, 2-chloro-4-iodopyridine, Figure 3, **10** (239 mg, 1 mmol) was added to the reaction mixture, which was then heated at 100 °C for 24 h. The mixture was then cooled, 10 mL water was added, and organics were extracted using dichloromethane (CH_2_Cl_2_). The CH_2_Cl_2_ layer was dried with anhydrous magnesium sulfate and purified using preparative TLC (hexane:ethyl acetate 1:1) to provide INFT, as an off-white solid **11** (30 mg, 0.1 mmol) in 10% yield. INFT: Mass Spectra (ESI): 322 [M + H]^+^ 100%; NMR (500 MHz, CDCl_3_): δ 9.66 (s, 1H), 8.38 (d, *J =* 5.3 Hz, 1H), 8.22 (d, *J =* 5.3 Hz, 1H), 7.88 (s, 1H), 7.59 (d, *J =* 3.45 Hz, 1H), 7.56 (d, *J =* 4.5 Hz, 1H), 6.74 (d, *J =* 3.4 Hz, 2H).

4-Chloro-2-(1H-pyrrolo[2,3-c]pyridine-1-yl)pyridine (ClNFT), **12**: was isolated as an off-white solid from the same reaction mixture as a side product (10 mg, 0.04 mmol). Mass Spectra (ESI): ClNFT: 230 (100%) 232 (94%); [M + H]^+^. NMR (500 MHz, CDCl_3_): δ 9.11 (s, 1H), 8.56 (d, *J =* 5.3 Hz, 1H), 8.42 (d, *J =* 5.3 Hz, 1H), 7.62 (s, 1H), 7.54 (d, *J =* 3.45 Hz, 1H), 7.47 (d, *J =* 4.5 Hz, 1H), 6.80 (d, *J =* 3.4 Hz, 2H).

4-Fluoro-2-(1H-pyrrolo[2,3-c]pyridine-1-yl)pyridine (FNFT), **13**: INFT, **11** (10 mg, 0.03 mmol, 0.2 mL DMF) was treated with tetrabutylammonium fluoride (0.2 mL, 1M THF solution) and heated at 120 °C for 90 min. The mixture was then cooled, 10 mL water was added, and organics were extracted using dichloromethane (CH_2_Cl_2_). The CH_2_Cl_2_ layer was dried with anhydrous magnesium sulfate and purified using preparative TLC (hexane:ethyl acetate 1:1) to provide a light brown oil, FNFT **13** (5 mg, 0.02 mmol). Mass Spectra (ESI): FNFT: 214 (95%) [M + H]^+^; 196 (100%); [214-F + H]^+^. NMR (500 MHz, CDCl_3_): δ 9.11 (s, 1H), 8.56 (d, *J =* 5.3 Hz, 1H), 8.42 (d, *J =* 5.3 Hz, 1H), 7.62 (s, 1H), 7.54 (d, *J =* 3.45 Hz, 1H), 7.47 (d, *J =* 4.5 Hz, 1H), 6.80 (d, *J =* 3.4 Hz, 2H).

### 4.3. Molecular Modeling

Using ChemDraw (ChemOffice version 21.0), energy-minimized molecular structures of INFT and IPPI were saved as mol files. To assess the binding of INFT to tau, we used the UCSF Chimera molecular modeling program as described previously for IPPI studies [14]. The reported cryo-EM three-dimension (3D) structure of tau fibril was used to perform molecular prediction on the tau fibril. The 3D model of tau fibril consists of the paired helical filament, which is the principal component of neurofibrillary tangles in AD with the characteristic appearance generated by a “double-helical stack of morphological units, each with a C-shaped cross-section displaying three domains”. This approach was previously used for the assessment of binding sites of IPPI.

### 4.4. Radiosynthesis

6-[^125^I]iodo-3-(1H-pyrrolo[2,3-c]pyridine-1-yl)isoquinoline, **8** [^125^I]INFT: To a solution of INFT, **11** (1 mg; 31 μmol) in anhydrous triethylamine (1 mL) under nitrogen, bistributyltin (60 mg; 103 μmol) and Tetrakis(triphenylphosphine)palladium(0) (5 mg; 4.3 μmol) were added. This reaction mixture was refluxed overnight at 90 °C. The dark yellow crude reaction mixture was purified over a prep silica gel TLC plate using hexane: ethyl acetate 1:1 as a solvent. The product was isolated as an oil (Figure 5, **14**) in a 40% yield. Mass Spectra (ESI): 482 (55%), 484 (100%), 486 (87%); [M + H]^+^.

A radioiodination hood (CBS Scientific, Inc., Escondido, California, USA) placed inside a fume hood designated to handle radioactive materials was used to carry out iodine-125 radiolabeleling of **14** using our previously reported methods [14,16]. The crude mixture was purified on preparative TLC (CH_2_Cl_2_:CH_3_OH 9:1) and separated from the unreacted starting material. [^125^I]INFT, **8** were separated and extracted using ethanol. RadioTLC of the ethanolic solution (Figure 5) showed purity >95% and Rf = 0.8, consistent with reference INFT. Ethanolic [^125^I]INFT, **8** at a 1 MBq/mL concentration, was used for in vitro experiments.

### 4.5. Human Tissue

All postmortem human brain studies were approved by the Institutional Biosafety Committee of the University of California, Irvine. Human postmortem brain tissue samples were obtained from Banner Sun Health Research Institute, Sun City, AZ, brain tissue repository for in vitro experiments. All AD brain, Parkinson’s disease and cognitively normal (CN) brain tissue samples were selected for end-stage pathology [1,15]. Human postmortem brain slices were obtained from chunks of frozen tissue on a Leica 1850 cryotome cooled to −20 °C.

### 4.6. Lipophilicity

1-Octanol 0.5 mL and 0.07 M phosphate-buffered saline (PBS), pH 7.4, 0.5 mL were presaturated, and [^125^I]INFT (118 kBq) was added. Similarly, to a separate set of 1-octanol-PBS mixture, [^125^I]IPPI (148 kBq) was added. The mixtures were vortexed for 2 min each, after which the tubes were centrifuged, layers separated and counted for iodine-125. Log D was computed from the activity levels in the octanol and PBS layers (average of *n* = 3 for each drug). 

### 4.7. In Vitro Postmortem Human Brain Autoradiography

Human anterior cingulate sections containing corpus callosum were sectioned from the subjects (AD, PD and CN). These sections were used to evaluate the effect of drugs on the binding of [^125^I]INFT to Tau. Unlabeled IPPI (10 μM) was used to measure nonspecific binding. The slides containing the sections (10 μm thick) were preincubated in PBS buffer for 15 min in eight slide chambers (one total binding and seven with the different drugs). The preincubation PBS buffer was discarded, and the appropriate amount of each drug (dissolved in ethanol) was added to the chambers with the slides. Each chamber was added [^125^I]INFT and 60 mL of 10% ethanolic PBS buffer for a final concentration of 3.7 kBq/mL of [^125^I]INFT. The chambers were incubated at 25 °C for 1.25 h. The slides were then washed with cold PBS buffer, 50% ethanolic PBS buffer twice, PBS buffer and cold water for 5, 5, 5, 5, 3 min, respectively. The slides with the brain sections were air dried, exposed overnight on a phosphor film, and then placed on the Phosphor Autoradiographic Imaging System/Cyclone Storage Phosphor System (Packard Instruments Co., Waltham, MA, USA). Regions of interest (ROIs) were drawn on the slices and the extent of binding of [^125^I]IPPI was measured in DLU/mm^2^ using the OptiQuant acquisition and analysis program (Packard Instruments Co.).

### 4.8. Immunohistochemistry

University of California-Irvine, Pathology Services used Ventana BenchMark Ultra protocols for immunostaining of brain sections. To determine the localization of Tau in the human AD brain sections, neighboring slices were immunostained with the DAKO polyclonal antibody, which binds to total Tau which detects all 6 six isoforms of Tau (dilution 1: 3000, A0024; Agilent, CA, USA). Immunostained sections were scanned using the Ventana Roche instrumentation and the images were analyzed using QuPath software version 0.4.3.

### 4.9. Image Analysis

Statistical differences between groups (AD, CN and PD) were determined using Microsoft Excel 16. Statistical power was determined with Student’s *t* test and *p* < 0.05 was considered to be significant.

## 5. Conclusions

In summary, a less lipophilic Tau imaging agent, [^125^I]INFT has been developed, which is suitable for autoradiographic studies of postmortem human AD brains. Further studies are planned to evaluate this new agent’s potential in vivo imaging value when labeled with iodine-124 for PET studies and iodine-123 for SPECT studies in transgenic mice expressing Tau. Possibility of using this less lipophilic azaindole backbone structure for potential fluorine-18 analogs for PET imaging of AD mice models of Tau will be investigated.

## Figures and Tables

**Table 1:** Binding affinity and lipophilicity of Tau agents.

Compound #	Name	Tau Affinity, IC_50_	cLogP	Radioligand/Tau Source	Reference
11	INFT	7.32 × 10^−8^	2.96 2.71 ^a^	[^125^I]INFT; Human AD brain slice	This work
12	ClNFT	2.22 × 10^−7^	2.56	[^125^I]INFT; Human AD brain slice	This work
13	FNFT	1.93 × 10^−7^	2.05	[^125^I]INFT; Human AD brain slice	This work
7	IPPI IPPI (K_i_)	8.43 × 10^−8^ 0.75 × 10^−9^	4.34 4.10 ^a^	[^125^I]INFT; Human AD brain slice [^3^H]; Human AD homogenate	This work [9]
4	MK-6240	0.36 × 10^9^	3.32 ^b^	[^3^H]; Human AD homogenate	[8]

^a^ Measured log D of [^125^I]INFT and [^125^I]IPPI using octanol-phosphate buffered saline partition; ^b^ Reported log D of [^18^F]MK-6240 in octanol-buffer [8].

## Data Availability

The data that support the findings of this study are available from the corresponding author upon reasonable request.

## Sample Availability

Compound sample information is available from the authors.
